# Supplementary material for: Impaired Awareness in People with Severe Alcohol-Related Cognitive Deficits Including Korskoff’s Syndrome: A Network Analysis
Source: J Clin Med. 2023 Apr 26;12(9):3139. doi: 10.3390/jcm12093139 (PMC10178999; doi:10.3390/jcm12093139)
Supplement: Supplementary file 1 [file jcm-12-03139-s001.zip › jcm-2309798-supplementary.pdf]

Supplementary materials

To test the reliability of the network and the associations, we re-ran the analyses (including imputation) 1000 times on a random subsample (75%) of the data and evaluated the selection frequency (i.e. occurrence in the sparsified network) and median partial correlation (i.e. association strength) of each edge (see Table 1). The higher the median partial correlation (both positive and negative) the stronger the association. The number of the selection frequency indicates the occurrence of an association in the networks (0-1000). For example, the association between ADL and iADL was present in 991 out of the 1000 networks that resulted on the 1000 subsamples of the data.

Table S1. Median partial correlations and selection frequency of the associations between the different nodes (variables).

Node1	Node2	Median Partial Correlation	Selection Frequency
ADL	IADL	0.316	991
AES	CPS	0.302	972
Mansa	PCRS	0.275	916
RISE	AES	-0.223	790
Age	LoS	0.217	723
NPI-Ag	NPI-Ap	0.194	591
AES	NPI-Ap	0.188	533
LoS	IADL	0.186	526
IADL	AES	0.180	480
NPI-Psy	NPI-Dep	0.170	390
RISE	PCRS	-0.159	297
LoS	PCRS	-0.156	291
Sex	Edu	0.155	290
BZ.	NPI-Dep	-0.153	234
ADL	CPS	0.152	229
LoS	Mansa	0.152	284
RISE	NPI-Ap	-0.151	230
PCRS	CPS	0.150	235
NPI-Dep	NPI-Anx	0.150	239
ADL	AES	0.146	192
AP.	AES	-0.146	161
Mansa	RISE	0.145	214
NPI-Dep	NPI-Ap	0.145	184
AD.	BZ.	0.1393	140
AD.	NPI-Dep	-0.139	150
BZ.	NPI-Ag	-0.134	117
AES	PCRS	0.130	108
